# Seasonal Algae and Nutrient Removal by Polyaluminum Chloride and Chitosan in a Drinking Water Reservoir

**DOI:** 10.3390/polym18172179

**Published:** 2026-09-07

**Authors:** Kechang Dai, Lixue Cheng, Zhenxiu Zhang, Lei Zou, Jiayu Wang, Qingji Zhang, Wenqing Shi, Lin Zhu

**Affiliations:** 1 Nanjing Water Group Co., Ltd., Nanjing 210002, China; dkc2026@126.com (K.D.); 13913856432@163.com (L.C.); 2 Nanjing Xinkaiyuan Engineering Industry Co., Ltd., Nanjing 210012, China; lengling888524@126.com; 3 College of Environmental Science and Engineering, Nanjing University of Information Science & Technology, Nanjing 210044, China; 13675105810@163.com (L.Z.); beyond1804404091@outlook.com (J.W.); wqshi@nuist.edu.cn (W.S.); zhulin0510420@126.com (L.Z.)

**Keywords:** coagulation, reservoir water sources, emergency response, algae removal, nitrogen and phosphorus removal, seasonal variation, polyaluminum chloride, chitosan

## Abstract

Emergency treatment of algal blooms in lake and reservoir source waters requires coagulants that remain effective under changing raw water conditions. This study compared polyaluminum chloride (PAC) and chitosan (CTS) in raw water collected from Yangku Reservoir during wet and dry seasons. Jar tests evaluated algal density, algal biomass, and nitrogen and phosphorus fractions across a 3~15 mg/L reagent-mass dosage range. Treatment performance differed between seasons. Mean algal density and soluble reactive phosphorus (SRP) removals in the wet season were 63.12% and 68.51%, respectively, and an apparent 70.05% decrease in measured NH_4_^+^-N concentration was also observed. Because the fate of dissolved inorganic nitrogen was not resolved, the NH_4_^+^-N decrease should not be interpreted as direct coagulative removal. Dry season water had higher algal density and a higher SRP/TP ratio. PAC maintained relatively stable algal biomass removal across seasons and showed stronger phosphorus removal, whereas CTS was more sensitive to seasonal changes in the raw water matrix. These findings support season-specific preliminary screening of coagulants while highlighting the need for residual-Al, pilot-scale, and process-mechanism validation before full-scale application.

## 1. Introduction

Lake and reservoir water sources are crucial for urban water supply in China. In recent years, under the impacts of agricultural nonpoint source pollution and domestic sewage discharge, eutrophication has occurred in some water source areas, accompanied by elevated nitrogen and phosphorus concentrations and occasional algal blooms [1,2,3]. During the dry season, reduced water volume and prolonged hydraulic retention time make algal overgrowth more likely, with algal densities in some reservoirs reaching up to 10^7^ cells/L [4,5,6]. Algae increase the operational burden on conventional water treatment processes, and some species release algal toxins that pose a direct threat to water supply safety [7,8]. When algal blooms exceed the capacity of conventional treatment processes, emergency treatment measures become necessary. Enhanced coagulation is one of the most widely used emergency treatment strategies [9].

Coagulation is a crucial step in both raw water pretreatment and conventional water treatment, effectively removing suspended particles and partial nutrients through mechanisms such as charge neutralization, adsorption bridging, and sweep flocculation [10,11]. Coagulants commonly used in water treatment can be broadly classified into inorganic coagulants and natural polymeric coagulants. Polyaluminum chloride (PAC) is the most widely used inorganic coagulant because it promotes rapid floc formation and remains effective over a wide pH range [12]. However, its application may result in residual aluminum in treated water and increase the difficulty of disposing of aluminum-rich sludge [13,14]. Chitosan (CTS) is a biodegradable natural polymer with non-toxicity. Protonation of the amino groups on its molecular chains generates positive charges, allowing it to remove algae and certain contaminants through charge neutralization and adsorption bridging [15,16]. Nevertheless, its coagulation performance is strongly influenced by water quality conditions, including temperature, pH, and the concentration of organic matter [17,18,19,20]. Despite its lower environmental burden, the performance of CTS under varying seasonal conditions has not been systematically evaluated.

Coagulation-based technologies have been widely investigated for algae and phosphorus removal, with previous studies mainly focusing on improving coagulation efficiency, optimizing operational conditions, and evaluating the performance of different coagulant systems. For example, Liang et al. [18] optimized enhanced coagulation parameters using chitosan combined with PAC for algal removal, Gou et al. [21] simulated emergency treatment strategies for cyanobacterial blooms in lake and reservoir drinking water sources, and Ji et al. [22] applied response surface methodology (RSM) to optimize PAC coagulation conditions. Seasonal variations in raw water quality are particularly important for actual lake and reservoir water sources. During wet seasons, increased inflow generally results in higher turbidity, whereas algal abundance is relatively low. In contrast, dry seasons are often characterized by elevated algal density and a higher proportion of dissolved nutrients. These seasonal changes in water matrix conditions may affect coagulant hydrolysis speciation, colloidal destabilization, floc formation, and settling behavior, ultimately leading to variations in treatment performance [23,24,25,26,27]. Therefore, evaluating the seasonal applicability of different coagulants is essential for developing more effective and adaptive emergency treatment strategies for reservoir water quality management.

Previous studies have mainly focused on optimizing individual coagulants or PAC-CTS composite systems under specific water quality conditions. In contrast, fewer studies have directly compared PAC and CTS as separate coagulants using real reservoir water collected under contrasting seasonal conditions. The present study therefore focuses on whether wet- and dry-season changes in the raw water matrix alter the relative performance of PAC and CTS. Using the same dosage gradient for both coagulants, we compared their effects on algae and nitrogen and phosphorus indicators and evaluated their season-dependent applicability as a preliminary screening approach for emergency reservoir water treatment.

## 2. Materials and Methods

### 2.1. Study Area and Raw Water Collection

Yangku Reservoir is located in the southwestern part of Hengxi Subdistrict, Jiangning District, Nanjing, Jiangsu Province, China. The reservoir primarily serves flood control and irrigation and also provides ecological water replenishment. In addition, it serves as an emergency backup drinking water source for the local area. Agricultural land and rural settlements dominate the surrounding area, resulting in considerable nonpoint source pollution input. Four representative sampling sites were established in the reservoir. The sampling area is located at 31.7° N and 118.6° E. Water samples were collected during the dry season (from February to April in 2024) and the wet season (from June to August in 2024), with three independent sampling events conducted at each sampling site. After collection, the water samples were transported back to the laboratory in a cooler at 4 °C. Coagulation experiments and water quality analyses were completed within 24 h. The available physicochemical characteristics of the raw water in April and August of 2024 are summarized in Table 1**,** including pH, dissolved oxygen (DO), and temperature.

### 2.2. Experimental Materials and Coagulation Experiment

Polyaluminum chloride (PAC; Sinopharm Chemical Reagent Co., Ltd., Shanghai, China; product No. 3934917033) and chitosan (CTS; Sinopharm Chemical Reagent Co., Ltd., Shanghai, China; product No. 69047436, batch No. 20201202, biochemical reagent grade) were used in this study. The PAC had an Al_2_O_3_ content of 28 wt% and a basicity of 70~75%. The CTS had a degree of deacetylation of 80.0~95.0% and a viscosity of 50~800 mPa·s. An average molecular-weight value was not specified in the available product information for this commercial CTS.

PAC and CTS were separately dissolved in pure water and 0.2% (*v*/*v*) hydrochloric acid solution, respectively, to prepare stock solutions at 2 g/L. One liter of raw water was placed in each beaker, and PAC or CTS stock solution was added to obtain reagent-mass dosages of 3, 6, 9, 12, and 15 mg/L. The PAC dosages are expressed as the mass of PAC reagent added per liter of raw water rather than as Al- or Al_2_O_3_-equivalent concentrations. Based on the 28 wt% Al_2_O_3_ content, the corresponding Al_2_O_3_-equivalent concentrations were 0.84, 1.68, 2.52, 3.36, and 4.20 mg/L, respectively.

The 3~15 mg/L range was selected as a low-to-moderate screening range based on previous jar test studies of PAC- and chitosan-based treatment of source and algae-containing waters, in which effective dosages varied from a few milligrams per liter to the low tens of milligrams per liter depending on the water matrix and coagulant system [22,28,29]. The same dosage gradient was used for both coagulants to enable direct comparison of their dose responses.

A multi-position magnetic stirrer (Model SN-MS-3D, Shanghai Shangpu Instrument and Equipment Co., Ltd., Shanghai, China) was used for coagulation. Samples were rapidly mixed at 300 r/min for 3 min and then slowly mixed at 100 r/min for 12 min. After 30 min of quiescent settling, the supernatant was collected for analysis. Each treatment was performed in triplicate. All coagulation experiments were conducted at laboratory room temperature on the day of sampling.

### 2.3. Sample Analysis and Data Processing

After homogenization, a 0.1 mL aliquot of the water sample was placed into a counting chamber. Under an optical microscope at 400× magnification, 20 random fields of view were selected to identify and count the cells of various algal genera, thereby calculating the algal density (cells/L). Algal biomass (mg/L) was estimated according to cell volume and by applying the conversion coefficient between biovolume and biomass. Nutrient analyses included total phosphorus (TP), soluble reactive phosphorus (SRP), total nitrogen (TN), ammonia nitrogen (NH_4_^+^-N), nitrate nitrogen (NO_3_^−^-N), and nitrite nitrogen (NO_2_^−^-N). Water samples were filtered through Whatman GF/F membranes prior to the determination of SRP, NH_4_^+^-N, NO_3_^−^-N, and NO_2_^−^-N. TP was measured using the potassium persulfate digestion-molybdate spectrophotometric method (GB/T 11893-1989) [30]; SRP was determined using the molybdenum antimony spectrophotometric method. TN was analyzed via alkaline potassium persulfate digestion-ultraviolet spectrophotometry (HJ 636-2012) [31]; NH_4_^+^-N was determined using Nessler reagent spectrophotometry (HJ 535-2009) [32]; NO_3_^−^-N was determined using ultraviolet spectrophotometry (HJ/T 346-2007) [33]; NO_2_^−^-N was determined by N-(1-naphthyl)-ethylenediamine spectrophotometry (GB/T 7493-1987) [34]. DO and pH were measured in situ using a dissolved oxygen meter (Model JPB-607A, Shanghai INESA Scientific Instrument Co., Ltd., Shanghai, China) and a pH meter (Model PHB-4, Shanghai INESA Scientific Instrument Co., Ltd., Shanghai, China), respectively. Water temperature was recorded from the dissolved oxygen meter corresponding to each sampling time.

The removal rate was calculated using the following formula: Removal rate (%) = (C_0_ − C)/C_0_ × 100%. C_0_ represents the concentration in raw water, and C refers to the concentration of supernatant after treatment. We used R software version 4.6.0 for data processing and plotting. Statistical analyses included Welch’s *t*-tests for seasonal comparisons, independent two-sample *t*-tests for PAC and CTS comparisons and paired *t*-tests for matched coagulation treatments. All statistical tests were two-sided. Exact *p*-values are reported in the text; values below 0.001 are reported as *p* < 0.001. The effects of different PAC and CTS doses were evaluated based on the removal efficiency trends. Due to the lack of sufficient independent replicates for each dose condition, statistical comparisons among individual dose groups were not performed. For pooled removal-rate comparisons, records with 2% ≤ removal < 100% were retained; values ≥100% were treated as below-detection-limit artifacts. The raw-water TN extreme value and five wet-season NH_4_^+^-N Below Detection Limit (BDL) values were excluded before seasonal testing.

## 3. Results and Discussion

### 3.1. Characteristics of Raw Water Quality During Dry and Wet Seasons

**Figure 1:**
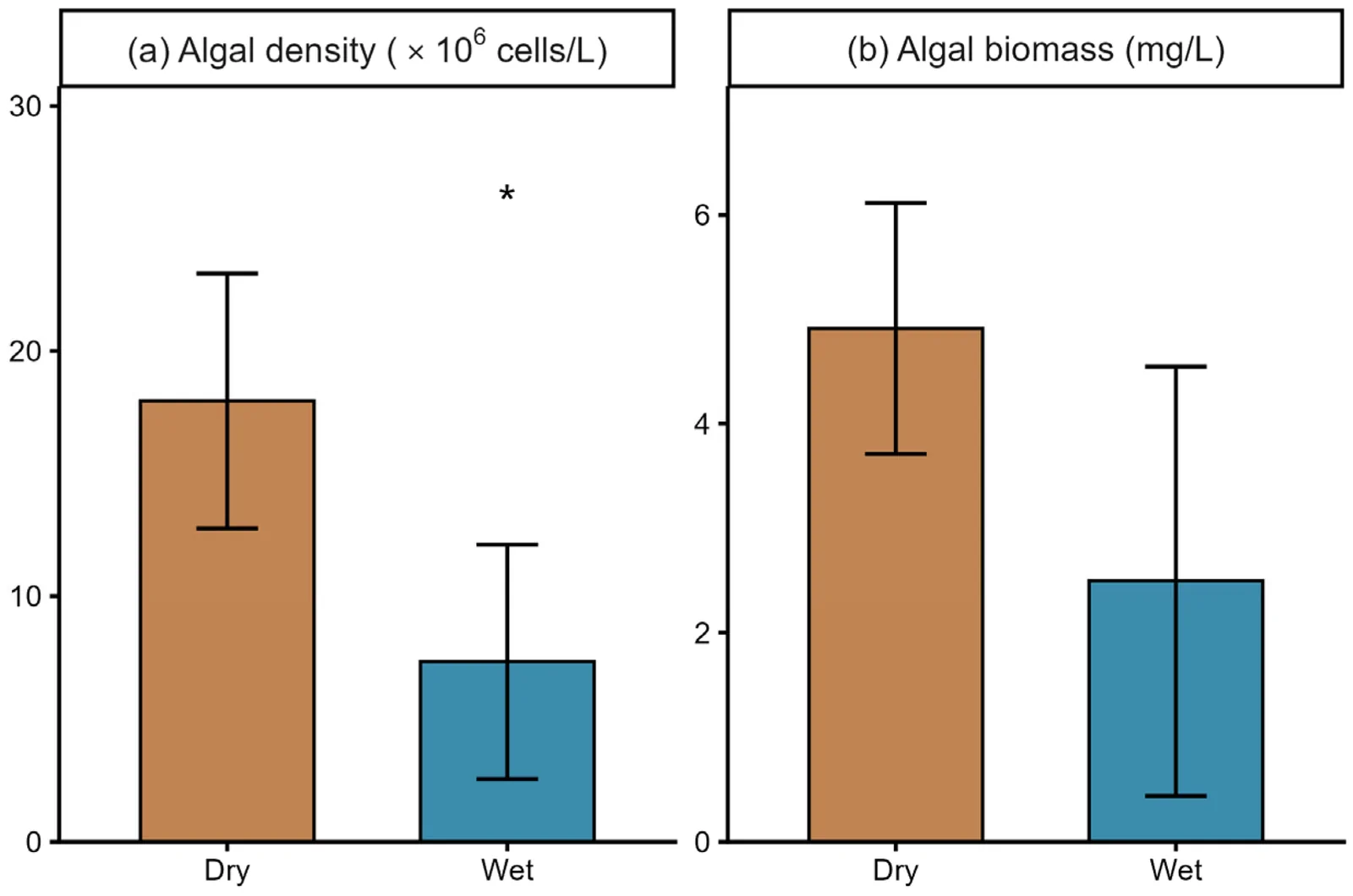
Seasonal comparison of raw water algal density and algal biomass: (**a**) algal density; (**b**) algal biomass. Algal density: *p* = 0.0405 *.

Raw water quality differed markedly between the two seasons (Figure 1). During the dry season, the average algal density was 17.96 × 10^6^ cells/L, with a relatively dispersed data distribution and a peak value of 23.25 × 10^6^ cells/L. In contrast, the average density during the wet season was 7.33 × 10^6^ cells/L, significantly lower than the dry season value (*p* = 0.0405). Regarding algal biomass, the average values were 4.91 mg/L in the dry season and 2.49 mg/L in the wet season. Both the algal density and biomass in the dry season were higher than those in the wet season. This phenomenon may be related to nutrient enrichment caused by reduced water volume and prolonged hydraulic retention time [5,7].

**Figure 2:**
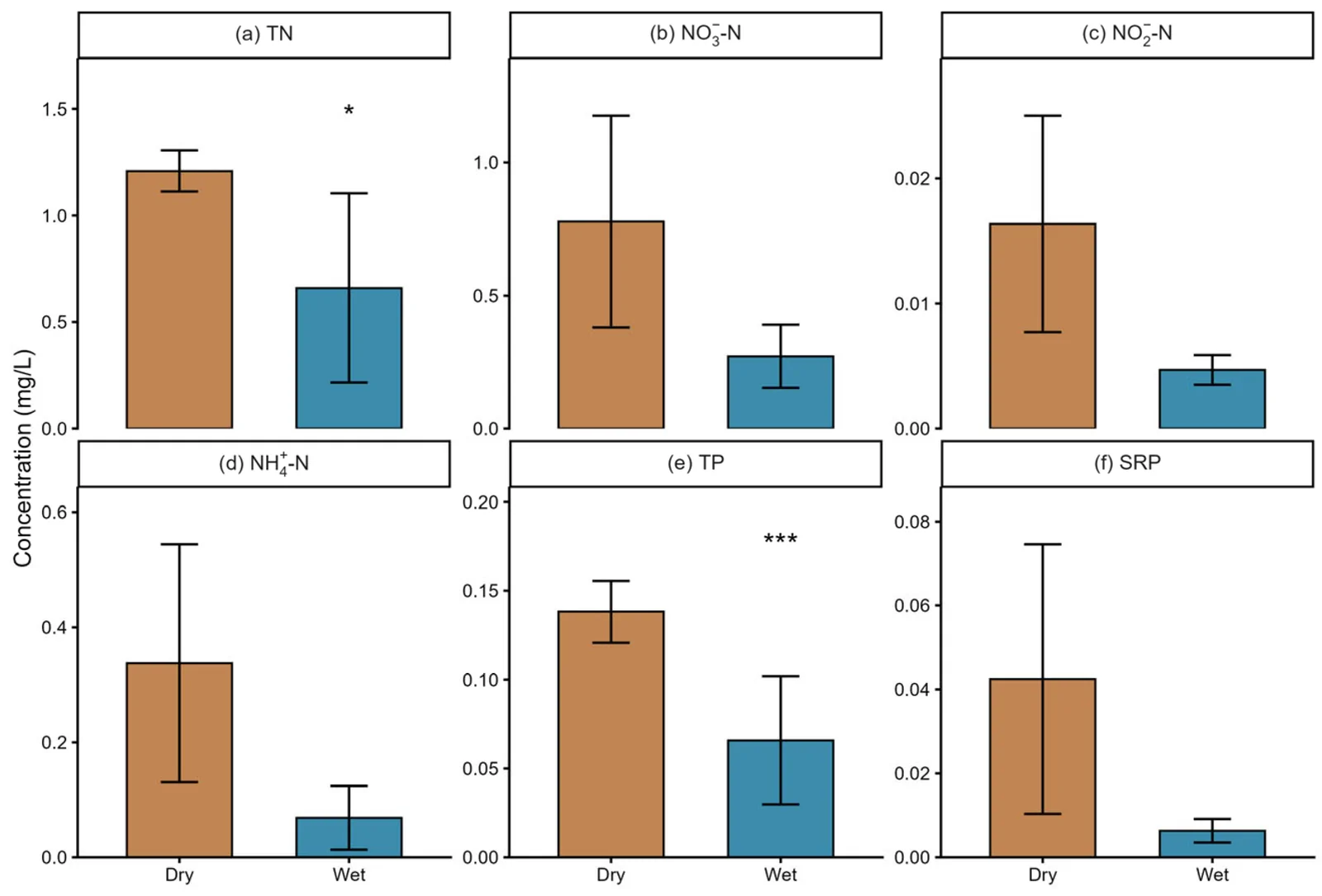
Comparison of nitrogen and phosphorus form concentrations in raw water during dry and wet seasons: (**a**) TN; (**b**) NO_3_^−^-N; (**c**) NO_2_^−^-N; (**d**) NH_4_^+^-N; (**e**) TP; (**f**) SRP. TN: *p* = 0.0166 *, TP: *p* < 0.001 ***.

Figure 2 presents the comparison of various nitrogen form concentrations in the raw water between the wet and dry seasons. During the dry season, the average concentrations of TN, NO_3_^−^-N, NO_2_^−^-N, and NH_4_^+^-N in the raw water were 1.21, 0.78, 0.016, and 0.34 mg/L, respectively. In contrast, the corresponding average concentrations during the wet season were 0.66, 0.27, 0.0047, and 0.069 mg/L. The average total nitrogen (TN) concentrations were significantly higher in the dry season (1.21 vs. 0.66 mg/L; Welch’s *t*-test, *p* = 0.0166). Similarly, dissolved inorganic nitrogen (DIN) concentrations were persistently greater during the dry season relative to the wet season. The lower proportion of measured dissolved inorganic nitrogen in wet-season TN suggests that a larger fraction of nitrogen may have occurred in particulate or dissolved organic forms [35,36]. These fractions were not measured directly in the present study. The higher proportion of DIN in TN during the dry season is consistent with accumulation under prolonged water retention, but the underlying transformation processes were not measured directly [37,38]. Dry season changes in dissolved organic matter (DOM) composition have likewise been shown to affect downstream water treatment performance [39].

The comparison of phosphorus form concentrations in the raw water between the wet and dry seasons is shown in Figure 2. During the dry season, the average concentrations of TP and SRP in the raw water were 0.14 mg/L and 0.042 mg/L, respectively, whereas those in the wet season were 0.066 mg/L and 0.0063 mg/L. The bioavailability of phosphorus was estimated based on the SRP/TP ratio [40,41,42], which was 0.30 in the dry season and 0.095 in the wet season, indicating a higher proportion of phosphorus directly available for algal utilization during the dry season. Since SRP is the phosphorus form that can be directly absorbed by algae, its increased proportion promotes algal growth [43,44], which also explains the higher algal density observed in the dry season. Seasonal differences in the particulate and organic matter matrix may have contributed to the observed coagulation differences. However, turbidity and organic-matter surrogates such as DOC, COD, or UV_254_ were not systematically measured in the present study; therefore, their contributions cannot be quantified directly. A study by Zhang et al. [45] on optimizing conventional processes under fluctuating water quality conditions also pointed out that seasonal water quality variations are a critical factor affecting the stability of coagulation performance.

### 3.2. Effect of PAC and CTS on Algae Removal

**Figure 3:**
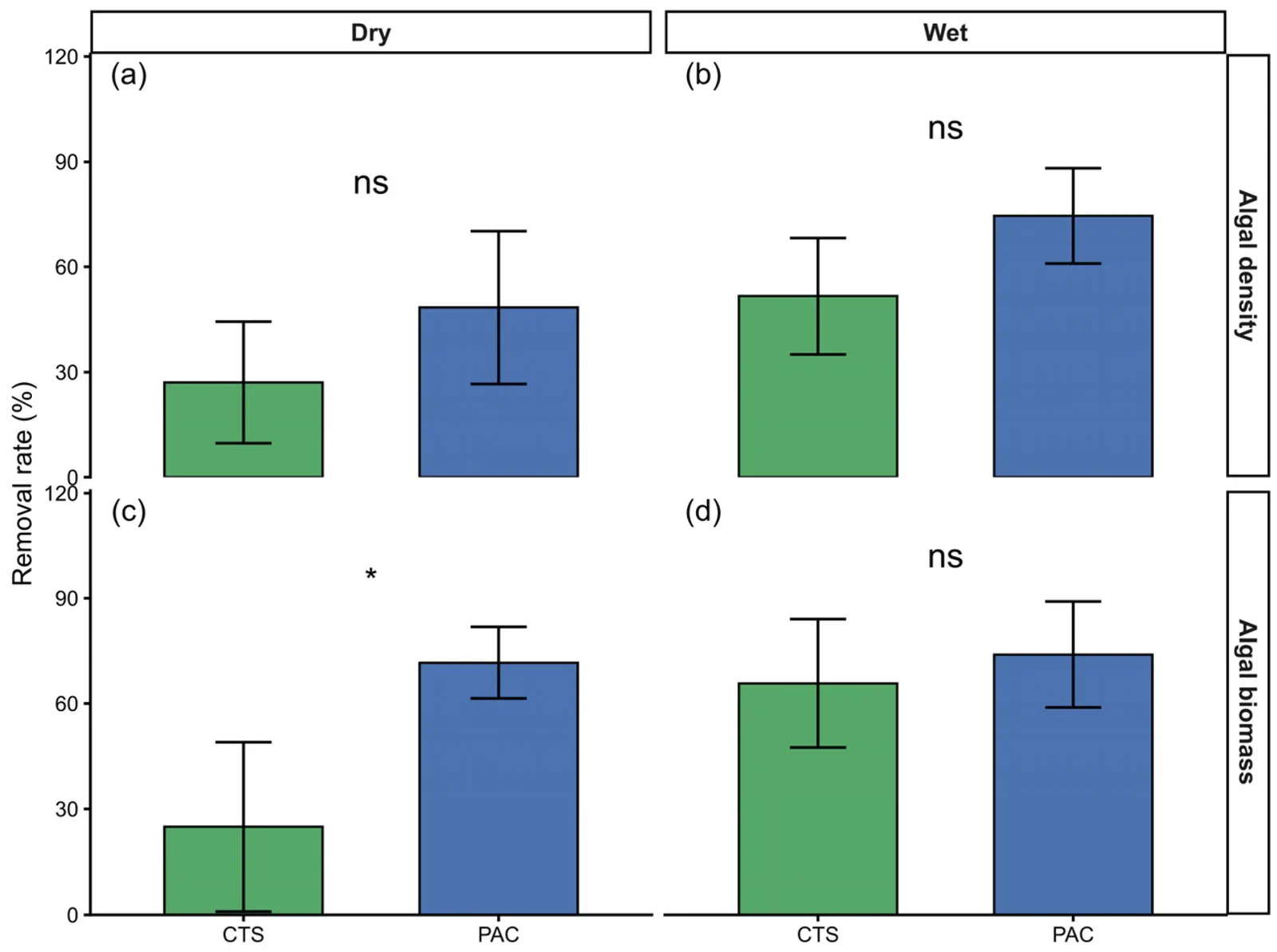
Algal removal by PAC and CTS in the dry and wet seasons: (**a**,**b**) algal density; (**c**,**d**) algal biomass. Dry algal biomass: *p* = 0.0111 *. The “ns” indicates not significant (*p* ≥ 0.05).

The comparison of the algal removal performance of the two coagulants between the wet and dry seasons is presented in Figure 3. During the wet season, the average algal density removal rates of PAC and CTS were 74.57% and 51.67%, respectively, while their average algal biomass removal rates were 73.96% and 65.74%, respectively. In contrast, during the dry season, the average algal density removal rates for PAC and CTS decreased to 48.42% and 27.07%, respectively, and their average algal biomass removal rates were 71.63% and 24.95%, respectively. Overall, the algal removal performance was superior in the wet season compared to the dry season (Figure 3). The average removal rates for algal density and algal biomass in the wet season were 63.12% and 69.85%, respectively, whereas those in the dry season were 37.74% and 48.29%, respectively.

PAC exhibited stable algal removal performance across both seasons. Even during the dry season, the algal biomass removal rate remained as high as 71.63%, showing only a marginal decrease of approximately 2 percentage points compared to the wet season. Upon hydrolysis, PAC generates various aluminum hydroxy polymers. These polymers compress the electrical double layer of algal cells through charge neutralization, while simultaneously forming aluminum hydroxide flocs to achieve sweep flocculation [46]. PAC hydrolysis products may contribute to floc formation when particulate carriers are limited, but turbidity and suspended-particle concentrations were not systematically measured in this study. PAC achieved 74.57% density removal across the tested dosage range in the wet season, higher than the roughly 60% cyanobacteria removal reported for a chitosan-modified kaolin (100 mg/L) plus PAC (10 mg/L) scheme in a simulated bloom emergency [21]. This improvement may be attributed to the lower background algal density in the raw water of the Yangku Reservoir compared to the severe bloom levels reported in the study by Gou et al. [21]. Furthermore, Ji et al. [22] optimized the coagulation process parameters of PAC using response surface methodology, which also confirmed that PAC maintains a stable algal removal performance over a wide range of dosages.

In the wet season, the algae removal efficiency of CTS did not reach the level of PAC: the average density removal rate was 22.90 percentage points lower (51.67% vs. 74.57%), and the average biomass removal rate was also 8.22 percentage points lower. In the wet season, CTS provided moderate algae removal without introducing aluminum through the coagulant itself. Liang et al. [18] pointed out that the algae removal effect of CTS combined with PAC-enhanced coagulation was better than that of CTS used alone. In this study, the algae removal rate of CTS used alone during the wet season was lower than that of the combined use, but it still produced measurable algae removal during the wet-season tests; no external acceptance threshold was evaluated. During the dry season, the algal removal performance of CTS declined significantly. The average removal rate of algal biomass dropped from 65.74% to 24.95%, a decrease of 40.79 percentage points, which was far greater than the 2.33 percentage point decrease observed for PAC during the same period. CTS relies on charge neutralization and adsorption bridging. The observed seasonal contrast may reflect changes in algal load and the raw-water matrix, although the specific contributions of particle availability and settling conditions were not quantified. PAC-CTS composite treatment has been reported to improve performance under some low-turbidity conditions [47].

Seasonal differences in the concentration and composition of algal-derived organic matter may influence coagulation performance. Extracellular polymeric substances (EPS) secreted by algal cells and extracellular organic matter (EOM) can occupy the adsorption sites on the CTS molecular chains, causing competitive adsorption and thereby reducing the capture capacity of CTS for algal cells. In this study, EOM refers specifically to the extracellular fraction of algal organic matter, whereas algal organic matter (AOM) represents the total organic matter derived from algae, including both intracellular organic matter (IOM) and EOM. Yue et al. [25] found that different types of organic matter significantly affect the coagulation of algal-laden water, with humic-like substances exhibiting particularly pronounced interference. Furthermore, a review by Li et al. [26] indicated that when the AOM concentration exceeds a certain threshold, both the destabilization efficiency of the coagulant and the settling performance of the flocs are inhibited; however, AOM concentration was not quantified in the present study. CTS does not generate the aluminum hydroxide sweep-flocculation phase characteristic of PAC. The present data therefore support a seasonal difference in performance, but do not establish that a specific settling limitation caused the observed CTS decrease.

Overall, algal removal rate is higher during the wet season than in the dry season. Beyond the inherent mechanisms of the coagulant itself, this difference is closely related to the raw water conditions. During the wet season, the initial algal load is relatively low. Seasonal differences in the raw-water matrix may have contributed to the observed performance differences, although turbidity and suspended-particle concentrations were not systematically measured. Representative field water temperatures were 23.18 ± 0.95 °C in April and 33.05 ± 0.97 °C in August. Temperature can influence coagulation by affecting water viscosity, coagulant hydrolysis, particle-collision frequency, and floc growth [48]. Lower temperatures generally slow particle aggregation and may reduce floc growth and settling, while pre-hydrolyzed PAC can retain coagulation capacity over a relatively broad temperature range [49,50]. CTS performance may also vary with solution conditions that affect polymer conformation, protonation, and interparticle bridging [51]. However, all jar tests in the present study were conducted at laboratory room temperature. Therefore, the field-temperature difference is reported as a seasonal source-water characteristic and cannot be treated as a controlled reaction-temperature explanation for the observed coagulation differences. Conversely, during the dry season, the raw water contains a larger algal population. Tiny cyanobacterial cells release EOM and disrupt coagulation reactions [26,52]. Changes in DOM may also influence coagulant demand and floc formation, although these characteristics were not quantified in the present study [26,53].

Under the tested jar test conditions, PAC showed greater robustness than CTS for algal biomass removal, particularly in the dry season samples. CTS nevertheless remained a potentially useful candidate under selected wet season conditions because it does not add aluminum as a coagulant component. For the application of CTS during the dry season, it is possible to further explore the possibility of its combined addition with PAC to leverage the advantages of the organic/inorganic composite coagulation system.

### 3.3. Effect of PAC and CTS on Nutrient Removal

**Figure 4:**
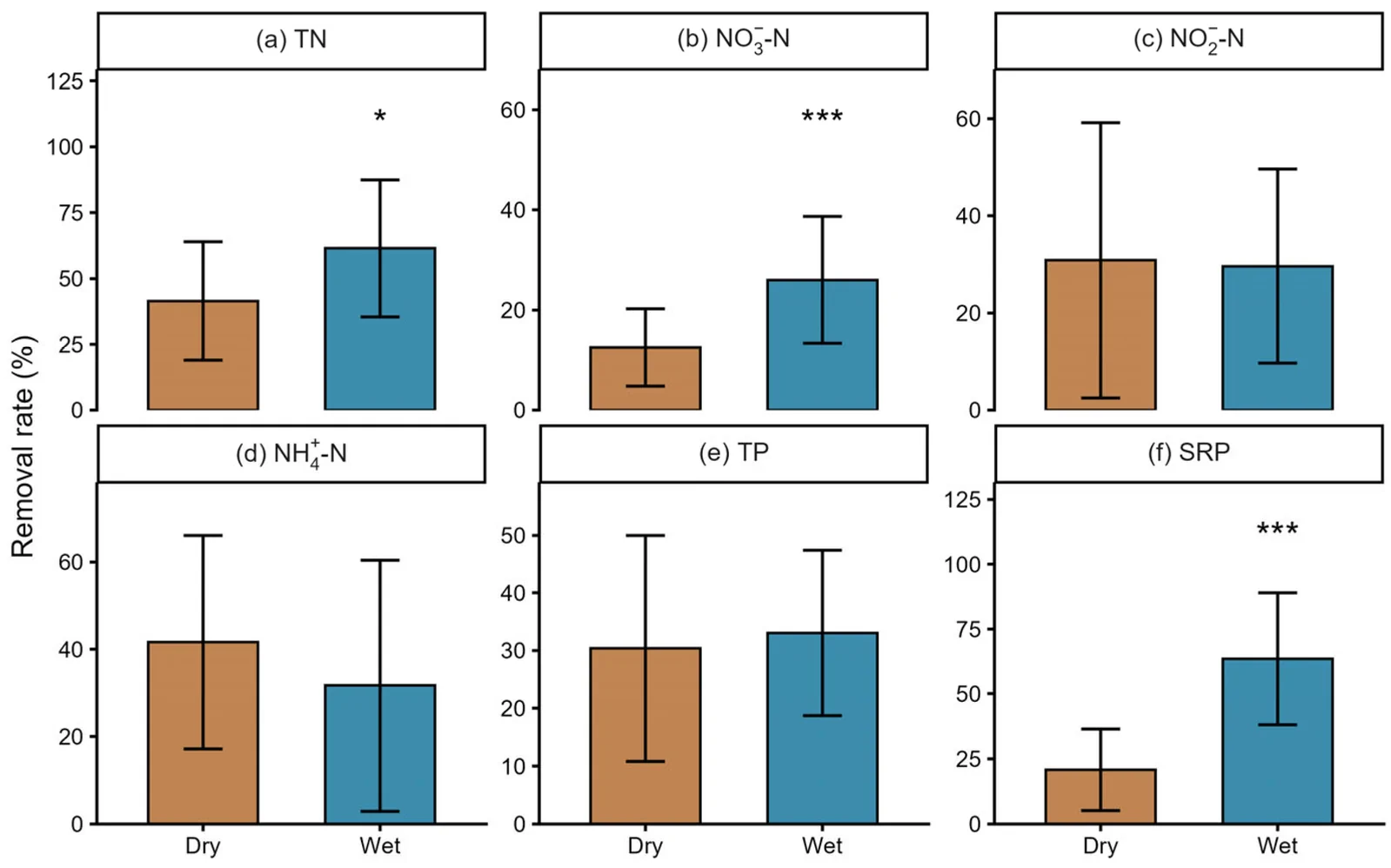
Seasonal comparison of nitrogen and phosphorus removal rates between the dry and wet seasons: (**a**) TN; (**b**) NO_3_^−^-N; (**c**) NO_2_^−^-N; (**d**) NH_4_^+^-N; (**e**) TP; (**f**) SRP. TN: *p* = 0.0141 *, NO_3_^−^-N and SRP: *p* < 0.001 ***.

The comparison of various nitrogen forms removal rates between the wet and dry seasons is presented in Figure 4. During the dry season, the average removal rates for TN, NO_3_^−^-N, NO_2_^−^-N, and NH_4_^+^-N were 51.05%, 20.85%, 38.60%, and 38.51%, respectively; whereas during the wet season, these values were 61.45%, 24.78%, 29.66%, and 70.05%, respectively. With the exception of NO_2_^−^-N, the removal rates of various nitrogen forms were generally higher in the wet season, with the most significant difference observed for NH_4_^+^-N. The lower proportion of measured dissolved inorganic nitrogen in wet-season TN suggests that a larger fraction of nitrogen may have occurred in particulate or dissolved organic forms. These fractions were not measured directly in the present study. The dry-season composition was relatively richer in DIN, but the specific sources and transformations were not resolved; coagulation is generally less effective for dissolved inorganic ions [35,54]. The average removal rates of NO_3_^−^-N were below 25% in both seasons. Because nitrate is a stable dissolved inorganic species, its concentration change cannot be attributed to direct conventional coagulation removal [54,55]. The approximately 70% decrease in measured NH_4_^+^-N concentration during the wet season jar tests is substantially higher than would normally be expected from coagulation alone. This value is therefore interpreted as an apparent concentration decrease during the experimental procedure rather than as evidence of direct NH_4_^+^-N removal by PAC or CTS. The present experimental design did not include process-specific measurements capable of distinguishing adsorption, biological or chemical transformation, analytical variability, or other concurrent processes. Consequently, no specific NH_4_^+^-N removal mechanism is assigned here. As an intermediate product of the nitrogen cycle, NO_2_^−^-N is chemically unstable, resulting in a certain degree of variability in its removal rate across both seasons.

**Figure 5:**
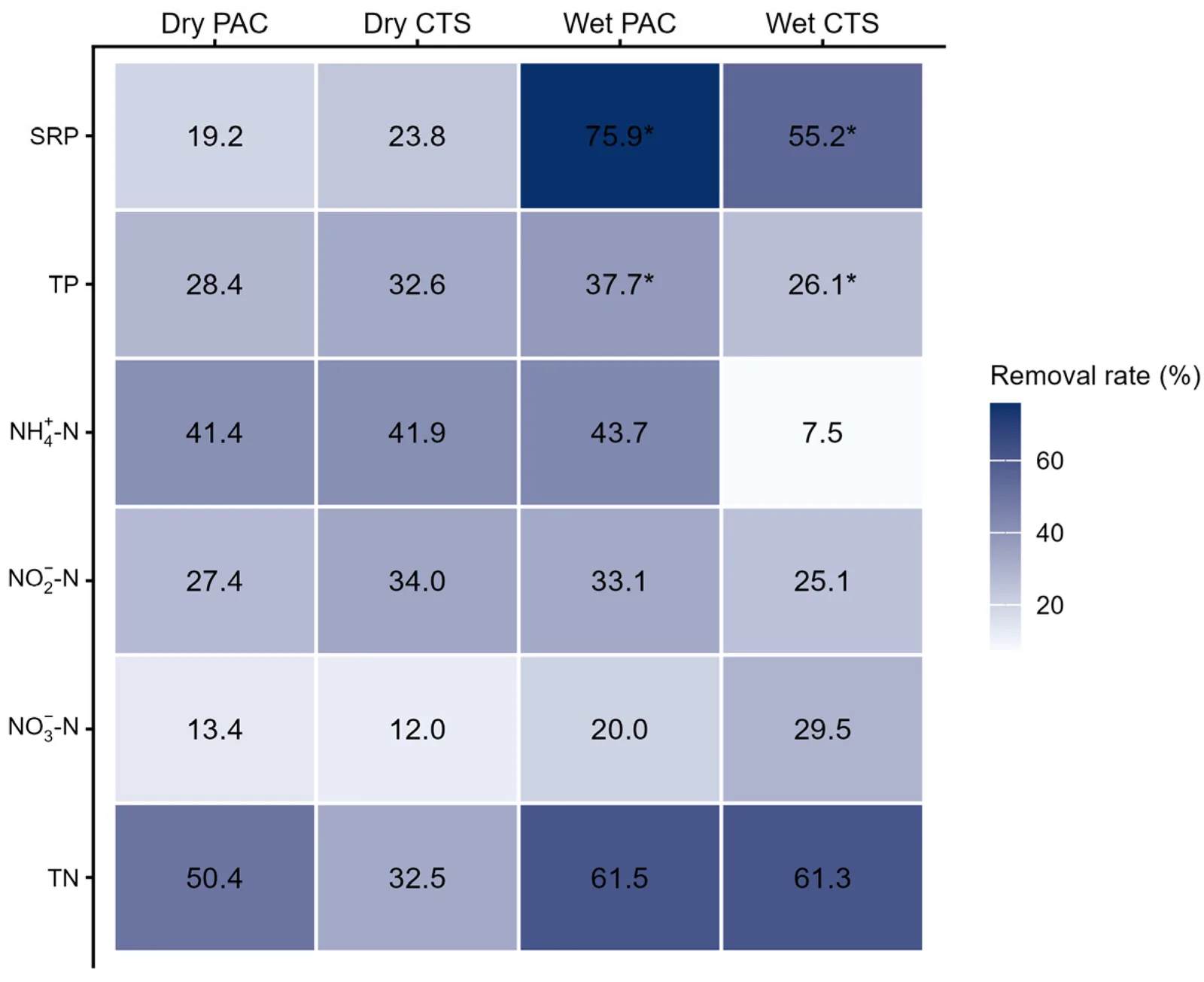
Heat map of the average removal rates of PAC and CTS for nutrient indicators during the dry and wet seasons. The values in each cell represent the average removal rates. Wet TP PAC vs. CTS: *p* = 0.0331; Wet SRP PAC vs. CTS: *p* = 0.0268. * indicates *p* < 0.05.

Regarding the differences in nitrogen removal between the two coagulants (Figure 5), PAC showed numerically higher or more stable TN performance under the tested conditions. During the wet season, CTS showed numerically similar TN removal to PAC. For NH_4_^+^-N, the observed seasonal contrast should be interpreted as a descriptive difference because the mechanism was not resolved. For NO_2_^−^-N, the relative numerical differences varied by season, without a statistically significant PAC–CTS difference. Overall, both coagulants showed limited efficacy in removing NO_3_^−^-N. CTS showed a numerically higher NO_3_^−^-N decrease during the wet season, which may be attributed to the adsorption potential of its active functional groups toward certain anionic nitrogen pollutants [56,57,58]. But the present experiment cannot determine whether this reflects direct coagulation, transformation, analytical variability, or another concurrent process.

The removal rates of different phosphorus fractions in wet and dry seasons are shown in Figure 4. The average removal rates of TP and SRP were 30.40% and 20.80% in the dry season, while they reached 33.06% and 68.51% in the wet season, respectively. The TP removal rate showed slight seasonal variation, whereas a distinct seasonal difference was observed for SRP removal. Phosphorus removal by coagulation is mainly achieved through three pathways: chemical precipitation between Al^3+^ and phosphate ions, phosphorus adsorption by hydrolysates, and enmeshment and sweep flocculation of particulate phosphorus [59,60]. The observed seasonal difference may reflect changes in the raw-water matrix, but the relative contributions of particle-associated capture, adsorption, and precipitation cannot be resolved because turbidity and particle concentrations were not systematically measured. The lower dry-season SRP removal cannot be assigned to a single mechanism from the present data. pH, aluminum hydrolysis and speciation, particle surfaces, and the organic matrix may all contribute, but their relative roles were not quantified. Lin et al. [61] reported a competitive relationship between phosphorus precipitation and organic matter destabilization in their research on cyanobacteria and phosphorus removal by electrocoagulation. This interpretation remains tentative because organic-matter concentrations were not quantified in the present study.

In terms of phosphorus removal performance between the two coagulants (Figure 5), PAC showed numerically higher SRP removal under the tested conditions. The PAC–CTS difference was significant in the wet season, whereas the corresponding dry-season comparison was not significant. Wet season SRP removal by PAC reached 82.81%. Although the efficiency declined in the dry season, it still remained higher than that of CTS. PAC provides Al-based precipitation and hydrolysis products that can contribute to SRP removal, but the relative contributions of these pathways were not quantified in the present experiments. Unlike PAC, unmodified CTS lacks an Al-based phosphate-precipitation pathway and therefore generally shows a lower capacity for SRP removal. Its contribution is expected to rely mainly on floc-associated capture and relatively weak adsorption interactions rather than metal-phosphate precipitation. As reported by Zhu et al. [62] in a review on phosphorus removal using chitosan-based adsorbents, unmodified chitosan has a low adsorption capacity for phosphate, and modifications such as metal ion loading are required to enhance its phosphorus removal performance. The lower dry season SRP removal cannot be assigned to a single mechanism from the present data. The dry season raw water SRP concentration was 0.042 mg P/L. Based on the PAC Al_2_O_3_ content of 28 wt%, the nominal Al:P molar ratio was already approximately 24:1 at 6 mg/L PAC and increased to approximately 61:1 at 15 mg/L PAC. Therefore, insufficient total aluminum availability alone is unlikely to explain the observed SRP behavior. Actual phosphorus removal can also depend on pH, Al hydrolysis and speciation, particle surfaces, and the organic matrix [28,63,64,65].

**Figure 6:**
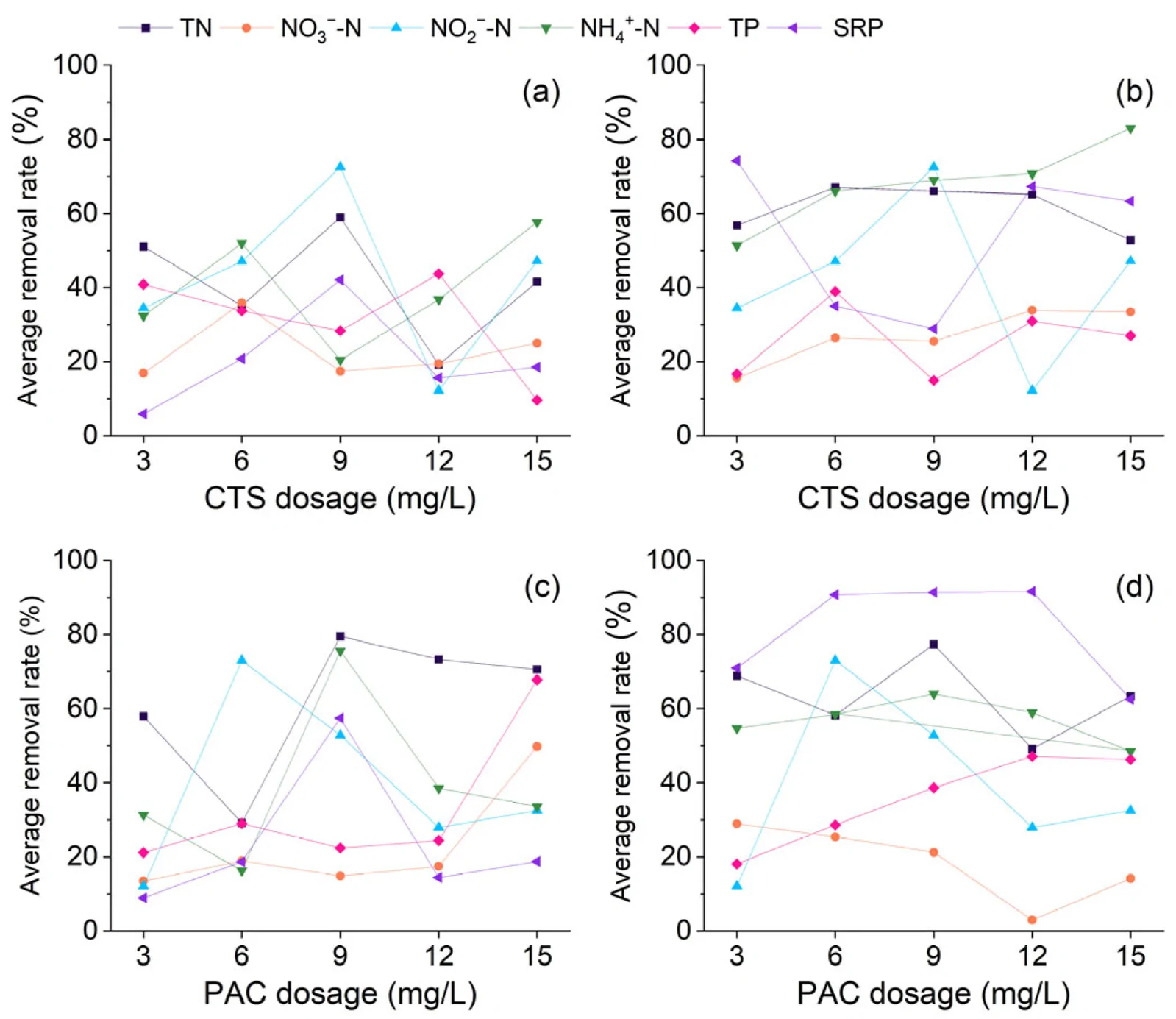
Dose–response relationships of nutrient removal by PAC and CTS in the dry and wet seasons: (**a**,**c**) dry season; (**b**,**d**) wet season. Each dose group (3, 6, 9, 12, 15 mg/L) corresponds to the average removal rate of the corresponding dose.

As illustrated in Figure 6, from the dose–response relationships of nutrient removal by the two coagulants, both PAC and CTS showed certain dose-dependent effects, but the responses of different nutrient indicators to the dosage of the coagulants varied. Overall, both coagulants could achieve good nutrient removal effects within the range of 6~12 mg/L. PAC showed better removal ability for phosphorus-containing nutrients, and the SRP removal rate remained at a high level within the range of 9~12 mg/L. At the same time, the removal effect of PAC for TP increased with the increase in dosage, and the TP removal rate reached a relatively high level at 15 mg/L during the dry season, indicating that increasing the dosage of PAC is beneficial for promoting the phosphorus removal process. This phenomenon may be related to the adsorption, complexation, and precipitation of phosphate by the Al(OH)_3_ flocs formed during the hydrolysis of aluminum salts [66,67]. At 6 mg/L PAC, the measured NO_2_^−^-N removal rate was 73.02% during the dry season jar test. However, because blank samples (without coagulant) were not included, this decrease cannot be attributed exclusively to coagulation, and transformation of NO_2_^−^ to other nitrogen species cannot be ruled out. In contrast, CTS showed better removal potential for some nitrogen-containing nutrients. During the dry season, CTS exhibited a relatively high removal efficiency for TN and NO_2_^−^-N at a concentration of 9 mg/L; during the wet season, CTS also demonstrated a stable removal effect for indicators such as TN and NH_4_^+^-N. However, as the dosage of CTS increased further, the mean removal rates of some indicators (such as TN and SRP during the dry season) showed a downward trend, which may be related to excessive charge neutralization, changes in the floc structure, or restabilization of colloidal particles [68,69]. The present data do not provide direct evidence for charge reversal or particle restabilization, so these mechanisms are mentioned only as possible explanations.

CTS has potential sustainability advantages because it is a bio-based and biodegradable polymer and does not add aluminum as a coagulant component. In some treatment systems, lower chitosan-based coagulant doses may also reduce inorganic chemical sludge. However, these benefits are not universal because the environmental footprint of CTS depends on raw-material sourcing, deacetylation and purification, acid consumption for dissolution, transport, and the dosage required for a specific water matrix [70,71,72,73]. Because no life-cycle assessment was performed in this study, CTS is discussed as a potentially lower-metal-input alternative rather than being quantitatively classified as the more sustainable option. Sludge production, dewaterability, composition, and disposal requirements were not quantified in the present jar tests. PAC is expected to generate aluminum-containing hydroxide flocs, whereas CTS does not add aluminum as a coagulant component; however, sludge volume and properties depend on the raw-water matrix and the captured algal and organic material.

## 4. Conclusions

This study compared PAC and CTS using real reservoir water collected during wet and dry seasons and demonstrated clear season-associated differences in coagulation performance. Under the tested jar-test conditions, PAC maintained relatively stable algal biomass removal and showed higher phosphorus removal, whereas CTS was more sensitive to seasonal changes in the raw water matrix. CTS nevertheless showed comparatively favorable performance for selected indicators during the wet-season tests and does not add aluminum as a coagulant component, although residual-metal performance was not measured.

The observed dose–response patterns provide a basis for preliminary seasonal coagulant screening, but they should not be interpreted as direct full-scale dosing recommendations. In particular, residual aluminum was not measured, and increasing PAC dosage cannot be recommended solely on the basis of phosphorus removal. Similarly, the apparent decreases observed for NH_4_^+^-N, NO_2_^−^-N, and NO_3_^−^-N should not be interpreted as evidence of direct coagulative removal because the fate of these dissolved nitrogen species was not resolved by the present experimental design. The present study was conducted as laboratory-scale jar-test screening using seasonally collected reservoir water; the observed performance and dose–response patterns therefore require continuous-flow pilot validation before any full-scale drinking-water application is considered.

Future work should evaluate PAC-CTS composite coagulation to determine whether PAC demand and aluminum input can be reduced while maintaining algae and phosphorus removal. Continuous-flow pilot validation under wet- and dry-season reservoir conditions, together with comprehensive assessments of residual aluminum, sludge characteristics, chemical consumption, operational stability, and treatment cost, is required before any potential full-scale drinking-water application can be considered.

## Figures and Tables

**Table 1:** Available physicochemical characteristics of raw water during the dry (April 2024) and wet (August 2024) seasons.

Season	Site	pH	DO (mg/L)	Temperature (°C)
Dry	1	7.98	5.45	24.5
Dry	2	8.20	6.00	23.2
Dry	3	8.07	6.21	22.6
Dry	4	8.67	7.05	22.4
Wet	1	9.38	18.45	32.7
Wet	2	9.60	14.67	32.6
Wet	3	8.62	8.32	32.4
Wet	4	8.79	10.86	34.5

## Data Availability

The datasets supporting the analyses presented in this study are available from the corresponding author upon reasonable request.

## Abbreviations

The following abbreviations are used in this manuscript:

PAC	Polyaluminum chloride
CTS	Chitosan
TP	Total phosphorus
SRP	Soluble reactive phosphorus
TN	Total nitrogen
NH_4_^+^-N	Ammonia nitrogen
NO_3_^−^-N	Nitrate nitrogen
NO_2_^−^-N	Nitrite nitrogen
DO	Dissolved oxygen
BDL	Below Detection Limit
DIN	Dissolved inorganic nitrogen
DOM	Dissolved organic matter
EPS	Extracellular polymeric substances
AOM	Algal organic matter (total algal-derived organic matter, including intracellular and extracellular fractions)
EOM	Extracellular organic matter (the extracellular fraction released by algal cells)
IOM	Intracellular organic matter (the intracellular fraction of algal organic matter)
RSM	Response surface methodology
